# Bilateral Breast Involvement in Multiple Myeloma: A Report of a Rare Case

**DOI:** 10.7759/cureus.70906

**Published:** 2024-10-05

**Authors:** Bano Alsaleh, Ahmed Alanzi, Mai Alsaeed, Abdulrahman Farahat, Mohammed Alsaleh, Hosameldin Mohamed

**Affiliations:** 1 Radiology, King Hamad University Hospital, Muharraq, BHR; 2 Anesthesia and Critical Care, King Hamad University Hospital, Muharraq, BHR; 3 Radiology, King Hamad University Hospital, Busaiteen, BHR; 4 Pathology and Laboratory Medicine, King Hamad University Hospital, Muharraq, BHR; 5 Pediatric Medicine, Mohammed Bin Khalifa Bin Salman Al Khalifa Specialist Cardiac Centre, Awali, BHR

**Keywords:** breast-radiology-mammography-ultrasound-mri-biopsyelastography, cancer, extramedullary multiple myeloma, metaplastic breast cancer, metastasis

## Abstract

Breast plasmacytoma is an uncommon extramedullary manifestation of multiple myeloma (MM). This case presents a primary extramedullary plasmacytoma and progression to MM with breast involvement after 12 years of discovering the disease. The patient is a 54-year-old woman who initially presented with a right humerus pathological fracture that turned out to be a primary extramedullary plasmacytoma. She received chemotherapy and achieved remission. Six years later, the disease recurred as a mass in the sternum, which was treated with radiation and chemotherapy but without improvement. The disease then progressed to MM with lesions in various bones. Following the first radiation treatment, the patient developed a new-onset cough, leading to a high-resolution CT, which incidentally revealed multiple bilateral breast lesions suggestive of malignancy. Mammography and sonography indicated multiple nodular densities warranting biopsy. Tru-cut biopsy confirmed plasmacytic neoplasm consistent with MM. Subsequent imaging showed the progression of breast lesions with increasingly aggressive features. Biopsies consistently indicated refractory progressive MM. This case highlights the aggressive, multisystemic nature of MM and the challenge of managing refractory disease with extensive systemic involvement, including rare bilateral breast lesions.

## Introduction

Multiple myeloma (MM) is a proliferative disorder of plasma cells, characterized by elevated levels of monoclonal paraprotein. It contributes to 1% of all cancer diagnoses and 10% of hematological malignancies [1]. The incidence of MM is slightly higher in men compared to women. The median age at diagnosis is approximately 70 years [2]. Due to improved disease management, the survival of patients with MM has increased considerably. Patients younger than 65 years have a 10-year improvement in survival rate; however, this trend is not consistent in patients aged above 75 years [3]. Unlike other cancers that spread to the bone, the osteolytic lesions in MM do not show any new bone formation. Bone disease is the primary cause of morbidity in MM [4]. However, MM can manifest in extraosseous locations either as solitary lesions or as a sign of MM relapse in some cases [5]. The involvement of breasts in MM is rarely documented in the literature. The present case discusses a case of breast involvement in MM.

## Case presentation

**Figure 1:**
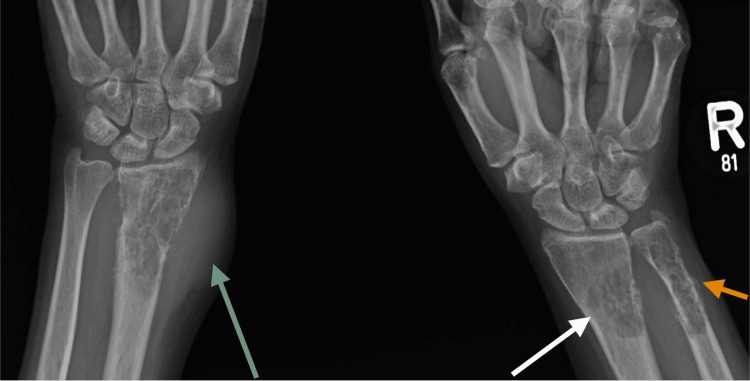
Bilateral wrist radiography in anteroposterior view Seen are lytic bone lesions with internal tribulation (white arrow) with moth-eaten appearance, involving the distal radius bilaterally and right ulna with cortical bone erosion and early destruction associated with periosteal reaction (orange arrow) and soft tissue component on the left wrist (green arrow). No fracture line or other similar lesions were observed.

A 55-year-old woman, who suffered a right humerus fracture 12 years ago, experienced an aggressive bone lesion suggestive of a pathological fracture following minor trauma. A subsequent biopsy revealed a plasmacytoma. She underwent autologous stem cell transplantation twice, initially responding well to treatment. However, six years later, she developed a large sternal mass indicative of disease progression, accompanied by body aches that showed multiple bone involvement with aggressive-looking lesions (Figure 1). Further investigation revealed multiple bone lesions consistent with MM. She underwent palliative radiotherapy, receiving a total of 30 Gy in 15 fractions, but post-treatment PET/CT showed persistent activity. A biopsy of the sternal lesion revealed amyloidosis, for which she was treated with lenalidomide and dexamethasone.

**Figure 2:**
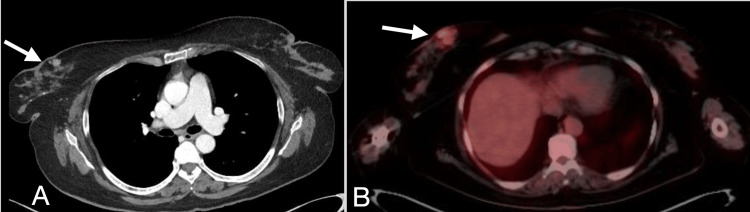
The CE-HRCT of the chest (A) and breast-level image from follow-up FDG PET/CT whole body scan (B) A: Observed are right mid-inner round non-calcific enhancing breast lesions, with few adjacent calcifications. B: Observed are multiple FDG avid right breast nodular lesions. The largest is seen at the mid-inner quadrants with SUVmax~5.4, appearing larger than in the previous chest CT (A), consistent with progressive course. CE-HRCT: Contrast-enhanced high-resolution computed tomography, FDG: Fluorodeoxyglucose

**Figure 3:**
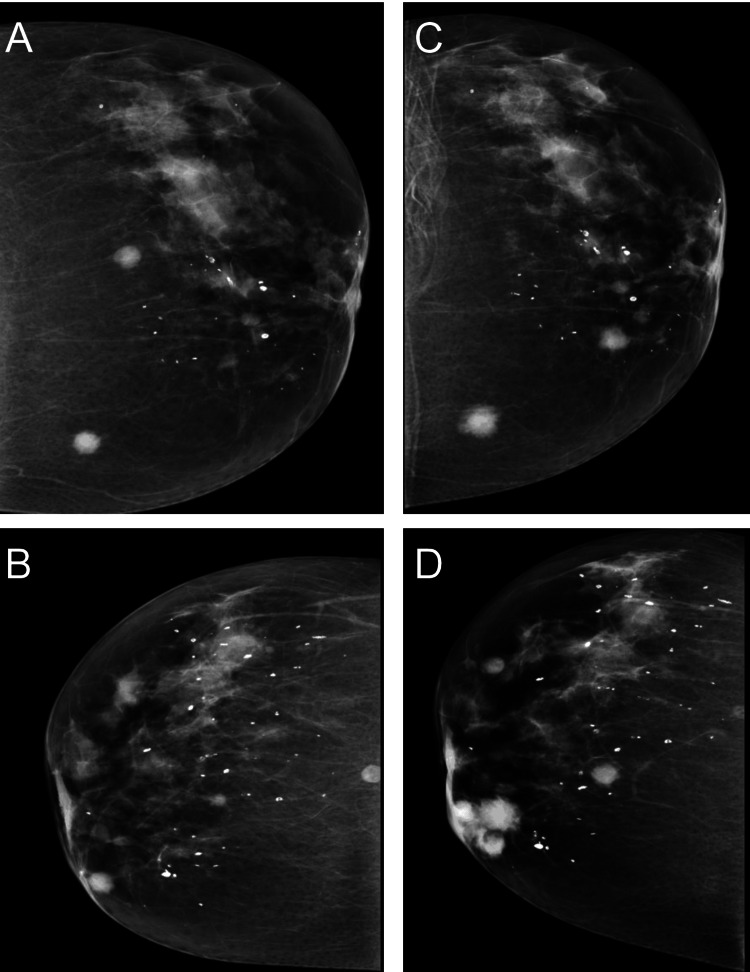
Bilateral mammography in craniocaudal views for ACR type c (breasts with heterogeneous fibroglandular density) A and B: Bilateral multiple scattered round circumscribed high densities with the bilateral multiple diffuse scattered as benign micro and macro calcifications. C and D: Interval increase in the size and number of the previously noted increased densities with architectural distortion mainly in the right lower inner quadrant with interval skin thickening. No skin or nipple retraction or lymphadenopathy was observed. ACR: American College of Radiology

**Figure 4:**
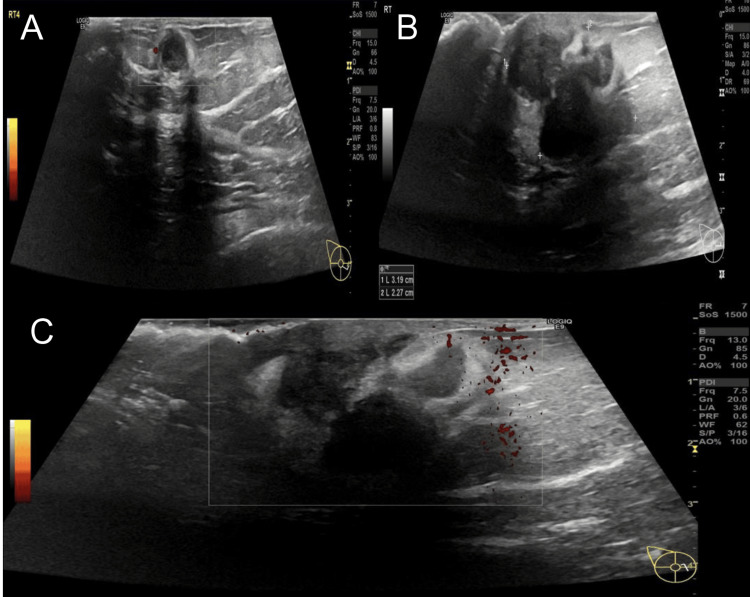
Right breast US of fibroglandular breast composition ACR type c A: Round-shaped, well-defined hypoechoic lesion seen in the right breast lower inner quadrant at 4 o’clock, with surrounding hyperechogenic rim, showing posterior acoustic enhancement. No internal vascularity was seen on the power Doppler. B and C: Amalgamated breast masses measuring about 32 mm x 24 mm (it was 11 mm x 10 mm) were seen encroaching the subareolar region along with posterior shadowing and overlying mild skin thickening and increased peripheral vascularity consistent with disease progression. No calcification, nipple or skin retraction, or lymphadenopathy were observed. ACR: American College of Radiology

**Figure 5:**
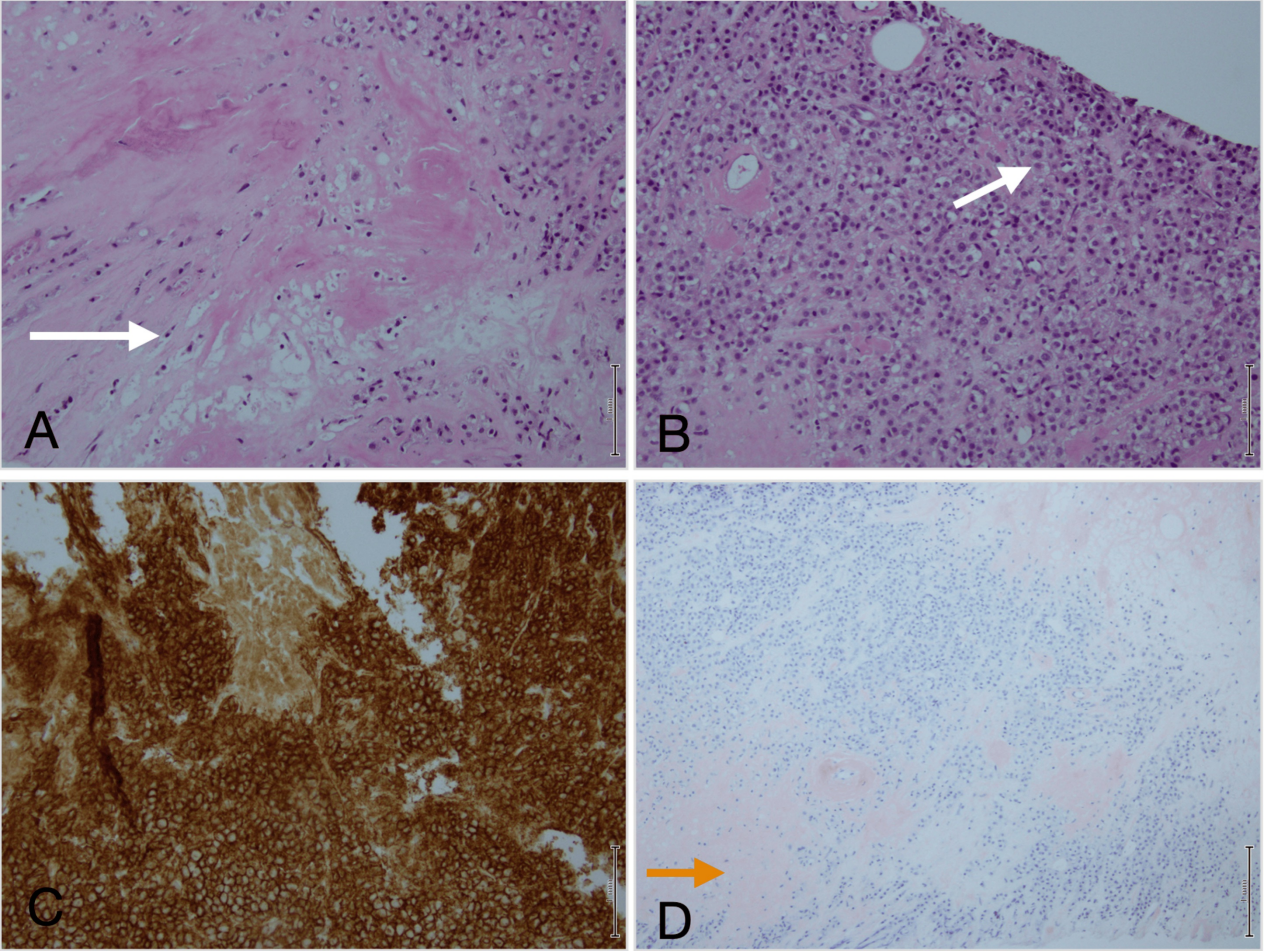
Histopathology tru-cut biopsy of the right breast lesion A and B: Cores of tissue are revealed to be infiltrated by sheets of neoplastic plasmacytoid cells (white arrow). There is deposition of amorphous amyloid material within the stroma and blood vessels. C: Immunohistochemistry shows the tissue to be positive for CD-138 D: A special stain of Congo-Red tests positive, denoting hyalinization and amyloid deposition (orange arrow).

The patient relapsed after multiple lines of treatment, including chemotherapy and autologous bone marrow transplantation. The patient was placed on maintenance chemotherapy. Palliative re-irradiation of the large sternal mass with 21 Gy in 7 fractions was followed. Then, she presented with a new onset of cough, for which a contrast-enhanced high-resolution computed tomography (CE-HRCT) of the chest was performed. The scan incidentally showed multiple variable-sized bilateral breast lesions (Figure 2) suggestive of malignancy for which further workup was advised. Bilateral mammography (Figure 3 A and B) and global breast ultrasonography (Figure 4 A) indicated multiple scattered hypoechoic round lesions with peripheral hyperechogenicity, warranting biopsy. Tru-cut biopsy confirmed plasmacytic neoplasm consistent with MM involvement (Figure 5).

Subsequent ultrasounds showed progression in size and number of both breast lesions, with increasingly aggressive features on imaging (Figure 4 B and C). Further biopsies were performed to rule out other malignancies, all consistent with refractory progressive MM. The patient was periodically examined through full-body or local diagnostic imaging in the outpatient department with no sign of recovery or regression observed. She continued to deteriorate with the continuous progression of the disease. She eventually died in 2024. 

## Discussion

The case presented is a rare example of a patient who initially developed a primary extramedullary plasmacytoma, followed by the progression of MM with bilateral breast involvement. The development of bilateral breast lesions is a rare and unusual manifestation of the disease. So far, less than 30 cases of breast involvement in MM have been reported [6]. Multiple myeloma is a hematological malignancy usually characterized by the clonal proliferation of the plasma cells of bone marrow with increased formation of the monoclonal immunoglobulins [7]. It commonly involves the chest wall, pleura, liver, lymph nodes, soft tissues or skin, central nervous system, lungs, genitourinary system, pancreas, and breast [8].

Multiple myeloma in the breast has been rarely documented. The first case of MM with breast involvement was reported in 1925 [9]. Most of the plasmacytes in the breast have been recognized in women with a mean age presentation of 53 years. It is also a disease of older adults, with a median age at diagnosis being 66 years. A few, 0.3%, 2%, and 10% of patients are younger than the ages of 30, 40, and 50 years, respectively [6]. Breast MM can be multiple or single. Bilateral and unilateral presentations have also been reported, with lesion sizes ranging from 1 cm to 7.5 cm. Furthermore, axillary lymph node involvement has also been reported in the literature [10].

These tumors may be present as solitary plasmacytic tumors without evidence of concurrent MM. An average treatment time of 1.5 to 2.5 years is required by 30% to 50% of extramedullary plasmacytoma cases to progress to MM [6]. In the present case report, the extramedullary plasmacytoma progressed into MM after 12 years. Additionally, most of the patients with MM in the breast present a palpable mass. A breast mass measuring about 32 mm x 24 mm is seen encroaching the subareolar region with overlying mild skin thickening and increased peripheral vascularity consistent with the disease progression as seen in this case report. The skin thickening and inflammation that occur can be confused with inflammatory carcinoma and breast abscess [11]. The differential diagnosis of such masses (presents within the clinical context of plasma cell neoplasm) includes primary epithelial neoplasm of the breast, pseudolymphoma, epitheliod malignant melanoma, non-Hodgkin’s lymphoma with plasmacytic features, and plasma cell mastitis [6].

The characteristics of MM in the breast are usually indistinguishable from other forms of breast disorders, either primary or metastatic. Thus, for plasmocytic tumors, the same imaging protocol (mammography) is applied to detect any suspicious breast mass. Multiple myeloma presents multiple or single, ill-defined or well-defined, and speculated mass lesions, often with microcalcifications representing some non-specific findings [12]. Bilateral multiple scattered round hyperdense mass lesions, heterogeneous fibroglandular density with bilateral multiple scattered round nodular densities, and bilateral multiple scattered benign with micro and macro calcifications were seen through mammography in the present case. 

Ultrasonic findings generally include well-defined hyperechoic and hypoechoic solid mass lesions. In our case, the US displayed bilateral multiple scattered rounds and ovoid hypoechoic lesions with surrounding hyperechogenicity, and Doppler US showed no significantly increased vascularity of these lesions. Breast plasmacytomas also demonstrated low-grade uptake of 18 fludeoxyglucose (FDG). The PET/CT showed multiple FDG-avid bilateral nodular lesions in the breast area in the present case [6]. As US and mammography findings are not adequate for diagnosis, the differential diagnosis for benign masses, other lymphoproliferative disorders, and primary carcinoma largely depends on histopathological evaluation [5]. So in our case, the diagnosis of bilateral breast plasmacytoma was confirmed by biopsy, which showed features consistent with refractory progressive MM. Lymph node dissection and surgical resection are also well-considered treatment options, but radiotherapy and chemotherapy are the most frequently employed options for breast MM [6].

In this case, the patient’s treatment plan was adjusted to include targeted therapy for refractory disease. The development of bilateral lesions with MM needs careful evaluation and consideration of alternative diagnoses. Moreover, this case underscores the challenge of managing refractory disease progression along with the adaptation of valid treatment plans.

## Conclusions

The present case describes the progressive course of MM in a 55-year-old woman, initially diagnosed following a pathological fracture. Despite undergoing multiple autologous stem cell transplantations and various chemotherapy regimens, the patient experienced recurrent disease manifestations. Further complications arose with the incidental discovery of multiple bilateral breast lesions, which, upon biopsy, confirmed infiltration by plasmacytic neoplasm consistent with MM. The rarity of breast involvement in MM is the main feature of this case that contributes to the existing knowledge on the topic.
